# Exploring health and disease concepts in healthcare practice: an empirical philosophy of medicine study

**DOI:** 10.1186/s12910-024-01037-9

**Published:** 2024-03-27

**Authors:** Rik R. van der Linden, Maartje H.N. Schermer

**Affiliations:** department of Medical Ethics, Philosophy & History of Medicine, Erasmus MC University Medical Center, Rotterdam, The Netherlands

**Keywords:** Health and disease concepts, Empirical philosophy, Pragmatism, Medicine, Healthcare, Philosophy of medicine

## Abstract

In line with recent proposals for experimental philosophy and philosophy of science in practice, we propose that the philosophy of medicine could benefit from incorporating empirical research, just as bioethics has. In this paper, we therefore take first steps towards the development of an empirical philosophy of medicine, that includes investigating practical and moral dimensions. This qualitative study gives insight into the views and experiences of a group of various medical professionals and patient representatives regarding the conceptualization of health and disease concepts in practice and the possible problems that surround them. This includes clinical, epistemological, and ethical issues. We have conducted qualitative interviews with a broad range of participants (*n* = 17), working in various health-related disciplines, fields and organizations. From the interviews, we highlight several different practical functions of definitions of health and disease. Furthermore, we discuss 5 types of problematic situations that emerged from the interviews and analyze the underlying conceptual issues. By providing theoretical frameworks and conceptual tools, and by suggesting conceptual changes or adaptations, philosophers might be able to help solve some of these problems. This empirical-philosophical study contributes to a more pragmatic way of understanding the relevance of conceptualizing health and disease by connecting the participants’ views and experiences to the theoretical debate. Going back and forth between theory and practice will likely result in a more complex but hopefully also better and more fruitful understanding of health and disease concepts.

**Supplementary Information:**

The online version contains supplementary material available at 10.1186/s12910-024-01037-9.

## Background

In the philosophy of medicine, scholars have primarily addressed ‘health’ and ‘disease’ as theoretical concepts without exploring their actual use in practice all too much. Yet, it has been argued that the way we conceptualize health and disease also affects the practical and moral dimension of medicine [1, 2]. While many philosophers recognize the practical consequences of defining health and disease in certain ways, most still tend to depart from theory to determine how health and disease should be defined. In the traditional analytical debate, only limited attention has been paid to the ways in which these concepts are embedded in the various practices they are deployed in. In the medical-philosophical literature, the conceptual, epistemic and bioethical issues associated with proposed disease-definitions, such as medicalization and overdiagnosis, have been primarily addressed as theoretical problems, often lacking contextualization and empirical foundation. Consequently, it is often not clear to what extent such conceptual issues are in fact experienced as problematic in practice and for *whom* exactly this is a problem. While it is increasingly recognized that the traditional method of conceptual analysis is ill-equipped to answer the various normative, ontological and epistemological questions surrounding the conceptualization of health and disease [2–4], new philosophical perspectives and research methods have to yet to be explored.

In recent contributions to the debate, several promising proposals have been made for a new direction, in which health and disease are viewed as plural concepts that need to be specified [4–11]. Instead of formulating definitions on monistic grounds, it is proposed to continue the debate by philosophical explication [4, 10], and by developing precising definitions [12]. This is important as concepts may serve various practical functions and are deployed in diverse contexts. As different practices may have different values, goals, and priorities, different types of definitions may be needed [7]. Moreover, we have recently suggested that we should assess the successfulness of concept definitions in relation to the function they serve in the context they are deployed in [5]. This shift towards a pragmatist stance requires scholars to look beyond theoretical arguments and to explore the various practical motivations of defining health and disease. Hence, when explicating concepts, it seems important to complement the theoretical debate by empirically studying the use of concepts in practice.

In contrast to the field of bioethics where empirical methods are commonly used to research attitudes, beliefs and perspectives of certain groups of people, empirical research is only seldomly conducted in philosophical studies on health, disease, and related concepts. Adding these methods to our philosophical toolbox enables us to investigate more closely how concepts of health and disease operate in medical practice and to explore what kind of problems occur in relation to them. We could use existing socio-empirical studies that, for example, investigate psychosocial and cultural aspects of certain diseases (e.g., see [13]), that review definitions and meanings of certain medical or bioethical concepts (e.g., see [14, 15]), or that explore patients’ and professionals’ views towards certain research programs or medical developments (e.g., see [16]). Both quantative and qualitative methods can be useful, depending on the research question at stake. However, as we propose in this paper, besides making use of existing empirical literature, we can also conduct empirical philosophy of medicine studies that aim to explore philosophical questions head-on.

Referring to debates on empirical ethics, Seidlein & Salloch [17] recently argued that the reconciliation of perspectives in the philosophy of medicine and socio-empirical research will lead to a more nuanced discussion that includes experiences of patients. Drawing on Alexander Kon’s [18] pragmatic classification of empirical methods, they argue that this approach may be used to investigate current practices (‘Lay of the Land’), revealing differences between illness conceptions in different groups of people, or between notions of ‘disease’ and ‘illness’. Such studies may improve patient-centered and shared decision-making, as it becomes clearer ‘what’ should be treated (cf. [19]). In addition to this, we argue that studying the views, attitudes and beliefs of medical researchers, clinicians and other healthcare stakeholders, seems important for obtaining a better and wider understanding of how health and disease concepts are used in actual practice and why they are conceptualized in certain ways. This proposal for incorporating tools and methods of the social sciences in philosophical work on health and disease concepts resonates with calls for experimental philosophy of medicine^1^ [20, 21], and for more ‘philosophy of science in practice’ [22, 23].

While there have not been many studies focused particularly on health and disease concepts in which empirical methods are used, some exceptions should be mentioned here. In Hofmann [24], physicians were presented a list of different conditions and were asked to classify them as disease or non-disease. Hofmann demonstrated that there are disparities between what physicians consider diseases. In Stronks et al. [25], lay people, randomly recruited on the streets, were asked to define what ‘health’ means to them. The study resulted in an extensive overview of different aspects of health and disease, categorized into multiple clusters, with interesting differences between socio-economic classes. In Kohne et al. [26], clinicians, patients, and clinicians who have been patients themselves, were interviewed to explore their ideas regarding the ontology of mental disorders. They observed that the ‘ontological palette’ is more diverse than is commonly perceived within the dominant scientific and educational discourse. In Van Heteren et al. [27], frontline professionals were interviewed to investigate their conceptions of health in clients with psychosocial problems. They observed that professionals define health in different ways but that they also accommodate for the views of their patients and to the broader context care is provided in.

As we understand health and disease concepts to be context-dependent, we believe it is important to investigate their function and problems arising in relation to them in various contexts. Regarding the methodology and the type of inquiry, our pragmatist approach encourages us to look for *problematic situations*. The term ‘problematic situations’ originates from the work of pragmatist John Dewey (see [28]), who argued that academic inquiry must always start with (solving) actual problems. Here, we will use the term problematic situation to describe as a situation in which current conceptions/definitions of health and disease are no longer sufficient for the continuation of a certain health care (related) practice, or the achievement of a goal of the specific practice that is at stake. Thus, besides mapping different health and disease conceptualizations, we primarily explore what kind of problematic situations are experienced in practice and investigate possible underlying conceptual issues. In doing so, we aim to further elucidate the philosophical debate on conceptualization of health and disease and give it more practical relevance. In this study we have therefore conducted qualitative interviews with a broad range of professionals and patient representatives, working in various health-related disciplines, fields and organizations. We chose qualitative methods because these are considered the most suitable for investigating new and underexplored areas.

## Methodology

### Design

We have designed a qualitative interview study with professionals working in various fields and organizations. Interviews were conducted by RL. As the sample included a broad range of professionals and patient representatives, a one-size-fits-all approach was not considered to be useful. We used a semi-structured interview guide that could be adjusted and specified to each of the interviews. This structure allowed us to explore context specific problems in more detail and to respond more extensively to issues participants mentioned during the interviews. Examples of interview questions include (for the complete guide, see appendix): ‘How would you describe ‘health’ and ‘disease’ yourself?’; ‘Would colleagues in your field agree with your definitions?’; ‘Are there any specific problematic situations that you encounter in practice that are related to definitions of health and disease?’; ‘Do you see any solutions to such problematic situations or have there been solutions brought forward to solve these issues?’. From these broader, more abstract questions, the interview was subsequently narrowed down to more specific questions, in response to the answers given by the participants. The interviews were conducted digitally, via Microsoft Teams, and took 46 min on average (ranging from 37 to 57 min). Audio recordings of the interviews were transcribed verbatim.

### Setting and recruitment

This study was conducted in The Netherlands. All participants were Dutch speaking and all were highly educated. All participants were selected following the principle of purposeful sampling. The reason for choosing for purposeful sampling was that we wanted to study definitions of disease and health in relation to actual problems arising in health-related practices. We recruited professionals who have spoken out in public or professionally about problems in relation to health and disease definitions and/or who work in fields/organizations that we considered to be interesting because we expected such issues to arise. Moreover, we aimed to cover a broad range of healthcare practices. The participants were recruited by e-mail.

### Participants

The sample details a broad range of professionals (*n* = 17), including doctors, policy makers, representatives of patient organizations, humanities experts, and medical professionals working in various advisory boards and governmental organizations (see appendix for a specified overview of participants their expertise). All participants were Dutch speaking, highly educated and experienced professionals. The representatives of the patient organizations that we included were interviewed in their professional role and not as patients (if applicable). One of the interviews had to be excluded from analysis because the recording was unusable due to a technological error, bringing down the total number of transcripts from 17 to 16.

### Data analysis

The data was analyzed using NVivo software (11th edition). The first interview reports and transcripts were discussed among RL and MS. Based on these discussions, RL made a first coding-scheme and discussed this with MS, which resulted in some adaptations. To reduce ‘tunnel vision’, transcripts were then analyzed and coded by RL and MS separately and compared afterwards. The interviews were analyzed in a way that may be best described as a method in between ‘grounded theory’ [29] and ‘directed content analysis’ [30]. That is, we did not build a conceptual scheme completely bottom-up as one would do with grounded theory. However, it was also not the case that we already had a solid theoretical framework at the start of the analysis which we would use to frame the issues discussed in the interviews, as is common in directed content analysis. We have taken the answers given by participants as a point of departure, exploring what their views are regarding the function of health and disease concepts, and exploring what kind of problematic situations they experience in practice. Sometimes, participants would already refer to specific theories, approaches or models themselves However, for other parts of the analysis, we have made use of distinctions and concepts from the academic literature to make sense of the many issues that were brought forward by participants. For instance, some issues mentioned by participants could be viewed as being practical examples of what is called a ‘line-drawing problem’ in the theoretical debate [10, 31]. Such categories appeared useful for analyzing and interpreting the data but where not selected prior to the analysis.

## Results

### Defining health and disease

In the interviews, respondents have pointed to various important practical functions of health and disease concepts. In some interviews the influence of certain definitions/approaches was explicitly articulated by participants. Participants talked about practical problems that they experienced and were often able to link these with how health and disease are conceptualized in their fields. For instance, some participants described specific models or definitions, such as the biopsychosocial model [32–34] and Positive Health [35, 36] and talked about their significance for their professional fields. In other interviews, however, the link between conceptualizations of health and disease and practical issues was more implicit. Participants would, for example, speak more broadly about ‘biomedical’ and ‘holistic’ approaches, or discussed how thinking in terms of ‘evidence based medicine’ (EBM) could (negatively) affect clinical practice.

While some of the respondents mentioned that it would be convenient to have general, all-encompassing definitions, none of them thought it would be possible to formulate them in a way that they are exhaustive and practically useful at the same time. Instead, in some interviews, viewing health and disease as plural concepts was discussed as being a possible alternative. HD01, says in this regard:

I’m not saying that one type of concept is primary or more legitimate than the other. But if you are talking about a health concept for the use in scientific research, then I would argue for a concept that is more clearly defined. If you’re talking about how people experience things or use, for example laymen, you could be talking about a simpler concept. And I think those things can coexist just fine.

At the same time, other participants were more hesitant when discussing the possibility of having multiple definitions of health and disease. Concerns were raised that such a situation may lead to problems of communication between institutions, (medical) disciplines, but possibly also between doctor and patient. As defining health and disease was viewed by many to be important to facilitate communication, for some participants it also seemed to be problematic to have a plurality of definitions. Furthermore, some participants would also critically question the endeavor of defining health and disease, questioning the goal of defining concepts itself. In several interviews, defining health and disease is described as a continuous process of reflection and adjustment, rather than a pursuit of finding ultimate answers. One participant, HD02, describes that how we define our concepts always have an effect on practice:

I think that every description is functional, in the sense that it always has an effect. Words aren’t neutral so it’s not- I don’t believe in that correspondence theory of there being something in reality that you just have to put the right term on. A word always does something. And I think that’s what it’s more about, so when use a certain view of health, for example, the absence of diagnosis. Then it is important to see, what effect does that have? Who or what is excluded? Or who benefits from this? Who gets worse from this?

### Health and disease concepts in practice

One of the key aims of this study was to explore how health and disease are conceptualized, defined or approached, in actual practice. In particular, we were interested what kind of practical functions health and disease concepts have in various contexts. In our analysis of the interviews, we observed that respondents discuss different types of health and disease concepts, working on different levels and as used for various kinds of purposes. If we look at the different type of functions and contexts the concepts are deployed in, and the levels on which they ‘operate’, an interesting picture emerges. We have categorized them broadly into three types of practical functions: (1) a ‘strategic, political and policy-making function’, (2) an ‘institutional and social function’, and (3), ‘guiding clinical practice and medical research’.

#### Strategic, political and policy-making function

In the context of strategic development, political debates and higher-order policy-making, definitions of health and disease can stay relatively broad and vague. Their function is not, for example, to give clinicians clear thresholds for line-drawing between the normal and pathological. Rather, their function is to steer public health policy, to change current practice within a healthcare organization, or to facilitate cooperation between organizations and institutions. Within this context, health and disease concepts do not need to have the analytical or explanatory power as may be needed in, for example, medical research or clinical practice. The definitions at stake may be demanding and idealistic, as they are used for questioning and/or changing the current state of affairs. Participant HD09 says in this regard:

If you want to explain to a politician why we are going to deploy all kinds of healthcare resources that are not directly focused physically, somatically, then you have to be able to explain it in clearly defined goals, objectives, and health definitions. And in that sense, it is of course also very important for the WHO to adjust such a definition. Because that changes your entire health policy worldwide. For example, it has an effect on what you use for prevention, but it also has an effect on what you use for treatment.

Embedded in these (inter)national discussions on definitions, goals and policies, we may find related discussions in the context of policy on local or organizational levels. Participant HD03 explains why defining health and disease concepts are considered to be important for organizational strategy and policy-making within healthcare organizations:

In the academic hospitals, we are primarily using a biomedical approach towards disease. At the same time, we have the ambition to expand to preventive medicine and to strive for positive health, public health, global health, that are all approaches of health. However, as an academic hospital you are only specialized in thinking about disease in biomedical terms.’’ … “So that’s the problem. If you make a strategy, what are you going to focus on? And so, what I say is, the wish is to focus on prevention, public health, global health and to look more broadly at health and disease.

Although broad and vague definitions may be used successfully for the purpose of guiding or changing policy, more concrete definitions may be needed in other contexts and for other purposes.

#### Institutional and social function

Another practical function that participants ascribed to the disease concept, and more concretely, to medical diagnosis, is a ‘gatekeeper function’ for issues regarding assessing eligibility for reimbursement of treatment and other healthcare arrangements. Examples mentioned by participants include debates on the legitimacy of viewing clinical conditions such as myalgic encephalomyelitis/chronic fatigue syndrome (ME/CFS) and chronic pain disorders as ‘genuine diseases’. What we consider to be diseases may therefore also be viewed to be a social and political agreement, some argue. Participant HD05 explains why ‘disease’ could be viewed as an institutional concept:

Who will be reimbursed for their medical treatment? That is decided on a political level.’’ … ‘‘And you can say that, at some point you have to say that someone has a disease, within the framework of a certain law, because that is how it has been agreed upon. And that is an institutional fact, because that is what has been agreed upon by various authorities.

What our institutions acknowledge as ‘genuine diseases’ does not only have impact within the medical realm, but also plays an important role in societal and personal debates. What we define as disease has also a social function. It creates a situation in which others take care of you as a patient, but it can also excuse responsibility from social tasks and duties, for example. In this regard, HD09 says the following:

And no matter how you look at it, sickness creates privileges. Because if you’re sick, people will bring you breakfast in bed, or not if you’re vomiting. And then you get get-well cards, people send flowers and you get time off. Then you are very pathetic and that comes with all kinds of perks. And I’m not saying that people get sick on purpose because of the perks, but that is an automatic consequence. Because my demented patients don’t get get-well cards and flowers and breakfast in bed at all, they are looked at strangely in the supermarket. And patients with psychiatric disorders, well, let’s say… they are usually not the most popular. And that has to do with the fact that we, I think, as a society have determined that being sick has to do with physical ailments…. There’s a discrepancy there. Physically ill: pathetic, perks. Not visibly ill: poser, difficult, hassle, hassle, hassle. That stings.

#### Guiding clinical practice and medical research

In a clinical context, health and disease can be approached in different ways depending on the type and level of care that is provided. For example, in emergency situations a medical doctor needs to focus on the direct biological problem, but when the patient is in a recovery phase they may have to ‘switch’ and take psychological and social aspects more into account. When caring for a patient suffering from a chronic condition a medical doctor may want to focus on aspects such as resilience and adaptation, and supporting the patient in what they consider to be meaningful. By going through these levels of care, health and disease may be approached differently. Here, HD06 explains this process of ‘shifting’ between models:

Of course, healthcare is very broad. The trauma surgeon and the emergency room doctor who provide acute care for a trauma patient, they are mainly focused on the biomedical model, their A, B, C, D, E, breathing, blood pressure, circulation, you name it. But then you end up in a rehabilitation process in which the biopsychosocial model is used. And then you come to an occupational doctor and an insurance doctor where I think it is very important to also use that model of Positive Health. Because there- Well, we see that with trauma patients too. In our research, independent of the seriousness of the injury, impediments to the ability to function were actually caused by all sorts of personal factors. So, you have to support people in finding their own direction and adaptability.

While taking account for ‘personal’ factors such as adaptability (or resilience) and societal participation may be of relevance for the treatment and revalidation of patients, and thus could be considered as being part of ‘health’, in context of medical research such factors are usually separated from health and disease outcomes and viewed as determinants instead. This allows researchers to measure causal relations between factors such as societal participation and health in a better way. Taking all kinds of (intra)personal and societal factors as *being part of* the health concept may result in problems for causal explanations in scientific research. Participant HD01 says the following regarding this tension:

The moment you use a broad concept of health, in which all these things are lumped together, you risk that the causality is not actually clear. So, in that sense, I’d like to stick to defining health as biomedical and mental functioning. And I would like to keep those other factors in their own place. And then you can look much better at, what causes what? Or how are things connected?

### Problematic situations in practice

A second key aim of this study was to ask participants if they did experience problematic situations in practice that are caused by or related to conceptual issues. In the interviews, a large variety of problematic situations were discussed, including various clinical, epistemological, and ethical issues. Some participants described more abstract problems such as ‘medicalization’ or ‘healthism’ in a broad sense, while others described more concrete issues, such as social or bureaucratic problems in case of patients with medically unexplained symptoms (MUS). Because of the diversity of participants included in our study (i.e., people working in different fields and organizations), the answers to our questions were also diverse and related to their particular context. We have clustered the problematic situations which were brought up in the interviews into 5 types:

1) Illness without identifiable pathology

2) Biomedical versus holistic approaches

3) Line-drawing and threshold problems

4) Problems with translational medicine: from research to the clinic

5) Communication problems

#### Illness without identifiable pathology

One issue that was discussed in several interviews is the problem of patients suffering from illness without identifiable pathology (or, ‘disease’). This includes patients suffering from ME/CFS, functional neurological disorders, chronic pain disorders, and other conditions that are often described under the umbrella term ‘medically unexplained symptoms’ (MUS). As illness is often viewed to be secondary to disease, and as it is commonplace to think that in order to overcome the illness, one has to cure the underlying disease, it seems only logical to search for the causing pathology. However, in many cases this search does not lead to a clearcut answer. As a result of this, unfortunately, the suffering of the patient is sometimes not taken seriously by medical professionals.

Besides being taken serious by medical professionals and getting the care they need, patients suffering from illness without known pathology may also encounter other type of problems. For example, for patients who cannot work due to illness a medical diagnosis is a necessary criterium to be met for being excused from work and to gain access to certain social and financial resources^2^. HD07 explains the institutional aspect of medical diagnosis:

Well, in this sense, we are dealing with legal frameworks. The law prescribes that to be able to claim a sickness benefit, one must be diagnosed with a disease. If it stops there, then we do not need to test those other two criteria. And sometimes you will find yourself in a gray area. Because yes, for example, I am also thinking about an example that I have. Social problems can also often lead to dysfunction. In the case of a social problem, there is not by definition disease, but can become one. And we often have to deal with those kinds of dilemmas, that if you see someone with informal care, with a financial problem, just to name a few- Those people who are walking on eggshells at a given moment when they come to us. We establish that, legally, there is no disease. But it might turn into disease.

In line with the situation sketched by HD07, HD15 argues that this problem of not getting recognized by our institutions as having a genuine disease, is a terrible experience for patients. HD15 explains that this in matter of fact urges their organization, a patient organization, to ‘medicalize’ the condition:

Then it will get very bad for them. Because people have a disease on the one hand, on the other hand, they always have to prove that they have it, and then there is also a financial need. So, that’s really the crux of the story. And, of course, we try with our work to make it clear as much as possible, that it is a progressive, biological condition, biomedical condition and that just needs research.

On the other hand, negative aspects of medicalization were also mentioned throughout the interviews. Participant HD14 mentions that including a condition in the ICD should be done with precaution:

The bottom line is that I’m a huge proponent of including pain in the ICD-11, the way as it is now. But I also see that there, I also see that in that balance of those arguments, there are, well, let’s just call it dangers. And that is that you do indeed have things that are normal part of life, which we are going to call disease. And that medical procedures are set up by people, who say, ‘hey, come to me, because I can solve it’. And that is, we have to be very careful about that, in communication, on the one hand to recognize that pain that is there, et cetera, and to take it seriously and with all the benefits that entails. But at the same time to ensure that we do not make it too medical where it is not desired.

In the interviews, many participants argue that, in clinical practice, the illness-experience of the patient is most important and deserves recognition. HD08 argues:

I think a disease is largely about the experience of the patient. And again, of course there is a biological construct underneath, but not always, eh. There are also people with a disease without a biological construct. And just to say, those people are not sick, I think that is far too short-sighted.’’ …. ‘‘We relatively often see people with a functional disorder, something that used to be called conversion or functional neurological symptoms. Those people can suffer a lot from this, but there is no biologically identifiable cause. And I think you shouldn’t dismiss those people as posers or say, you have nothing. No, they do have something and they do suffer from it and that leads to hindrance in daily life. So, I think you can speak of disease.

#### Biomedical versus holistic approaches

A broader issue that came up in many of the interviews is one that may be best described as problems that are due to biomedical versus holistic approaches towards health and disease. Participants discussed that focusing treatment primarily on a biomedical parameter while paying less attention to the experience of the patient as a whole can be problematic for providing good clinical care. That is, patients may be treated for their medical condition without taking sufficient account of their personal circumstances and/or life goals. Participant HD11 said in this regard:

Of course, you can approach disease in many different ways. If you approach it cell-chemically, so to speak, disease is what damages, or attacks, or if you will, the biochemical integrity of your cell. But if you look from a patient’s perspective, or from a doctor’s perspective, then a disease is something that hurts, bothers, hinders that patient. And the perspective of the patient, but also the approach of society, of course, plays a very important role in this.

In some cases, the emphasis on the biomedical paradigm may even lead to instances of ‘treating’ biomarkers that may not have a clear clinical significance. HD11, discussing the implications of the new drug (aducanumab) for Alzheimer’s Disease, explains that:

The bottom line is, there is a new drug that, if you look at the cellular level, biochemical level, it absolutely does something. It does something to the proteins in your brain, period. However, if you look at the clinical effect on the patient, and what it can do for the patient, it does nothing. Patients don’t improve, we have no improvement, cognition does not improve, general daily activities neither, nothing. The FDA has approved it on the grounds that, despite the fact that it doesn’t do anything clinically, biochemically the evidence is so clear that it does something, it’s bound to do something clinically. While it just doesn’t.

Yet, also in cases where a biomedical treatment has proven to be clinically effective, it could be nevertheless problematic to forget about the patient’s personal circumstances. Sometimes it may be more important to help people with psychosocial issues, for example, than to direct attention to the medical problem. Participant HD10 discusses person-centered care for diabetes patients and argues that taking care of the patient - improving their health - includes more than treating the disease biomedically:

That also touches on the need for person-centered care, - that the care providers really can actually see from the patient’s eyes which approach they should take. Do they really have to focus on that disorder or do they indeed have to focus on the social realm?

Another related problem that was mentioned in the interviews is that of prioritizing biomedical diagnosis over other holistic aspects when assessing the prognosis. Although the diagnosis may give important information regarding the development of a disease and about chances for successful treatment, other non-medical factors may have an underestimated influence on the prognosis as well. In some instances, psychosocial aspects may even show a stronger correlation with prognosis and treatment than the medical diagnosis does, participant HD04 says in this regard:

The classic assumption is very much like, if you know a diagnosis, then you know the prognosis and then you know whether or not you need to do something to influence that prognosis. Whether or not you can do something to influence that prognosis. And what we are gradually noticing is that that prognosis may well be determined by many other factors and that the diagnosis is only a small part of it and therefore only partly determines what the prognosis is. The prognosis is also determined by all kinds of other factors. other variables, to put it in scientific terms.

According to HD04, it is common for medical professionals to focus too much on biomedical diagnosis and to underestimate the influence of ‘non-medical’ variables on the prognosis and the well-being of patients – which, she beliefs, should be the ultimate aim. This does not only go for patients with medical unexplained symptoms, for which finding the right diagnosis is considered to be very difficult. Also for diseases that can be diagnosed straightforwardly there seems to exist a disparity between a biomedical view of disease and more holistic ones. HD04 gives the following example:

Examples abound. People with rheumatoid arthritis, we can diagnose rheumatoid arthritis fairly well with lab tests, with clinical tests, with imaging tests. We have criteria, you can always argue about that, but we generally agree on that. And then we also have a measure of the disease activity. So, if you have a very high sedimentation rate, then you have a high disease activity, for example. And if you then look at the severity of the complaints and the disability that people have and relate that to disease activity, then that is not a nice linear relationship. So, then there are people with, if you would look at it as a rheumatologist, as a doctor, if you look at it as a doctor, then well, that disease is just well under control, hardly swollen joints, no increased sedimentation rate, goes well, but in fact people suffer very much.

#### Line-drawing and treatment threshold problems

In the interviews, problems with drawing the lines between states of health, disease, or ‘being at-risk’, and problems with determining the right thresholds for starting medical interventions, were considered important reasons for having clear definitions. Having clear cut-offs for diagnosing disease and for starting treatment is seen as convenient for clinical practice. Participants expressed a desire to have objective measures to decide whether we are talking about disease, and when to start treatment. Yet, they were also highly doubtful if such clear lines could be drawn. On the one hand, they said diagnostic tests are used to examine if a patient deviates from the (objective, biomedical) norm. On the other hand, participants also argued that patients’ symptoms should be viewed as central to drawing the line. This also seems to be problematic, however, as patients may sometimes deviate from the norm but do not experience symptoms, or vice versa, patients may experience symptoms but test results do not show significant abnormalities. HD08 talks about the challenges of the line-drawing problem for clinical-decision making:

Of course, it is difficult, because doctors like to work neatly, like to work according to scientific evidence, like to work according to guidelines. And a guideline only works well if you can make hard statements, otherwise you have a guideline that says about everything: you ‘may consider this’. And yes, that is how guidelines end relatively often, but then it is not very useful in practice, because you want such a guideline to guide you. And the surgeon, just to name one, who wants to determine whether he should operate. And it’s easy if that just has a cut-off point that says, you have to operate above 23 and not below, just to name something. So, whenever there’s a big gray area, it’s complicated and leads to subjectivity and also different doctors making different decisions.

This was also discussed in relation to prevention, when patients are ‘treated’ with medication to prevent future disease(s) while they do not experience symptoms at that point of time. In particular, participants pointed to the lowering of diagnostic and treatment standards for risk-factors such as high blood pressure and high cholesterol as examples in which it is difficult to draw the line. Participant HD09, who reflect on this problem, says the following:

But you can get quit some muscle cramps from cholesterol lowering drugs. Yes, so it may be that he has one in twenty, one in thirty less chance of that stroke, but in the meantime, he is no longer able to walk down the stairs and do his own shopping because of those muscle complaints and perhaps even take a fall. Well, and it’s not the case that everyone has muscle problems, so for the people who don’t get this it might be the best treatment. That is the way you have to look at it. And also evaluate, eh, and that’s about when you start something, you have to follow up what it does to someone, even if someone has been using it for some time, because that can change.

When participants were asked if they could identify reasons for this trend of lowering diagnostic and treatment thresholds, some suggest that cultural values and norms play an important role. Not only there is an increasing societal pressure on living a healthy life, health is also increasingly viewed as a moral good. It is this normative shift, in combination with ever growing technological possibilities, that is suggested to lead medicine to focusing on early detection and treatment of health risks more and more – even if chances of developing actual diseases are expected to be low. Patients may desire more diagnostic testing or more frequent health check-ups and medical professionals may feel obliged to grant their requests, since the technology is available. This is not without consequences, however. HD11, for example, explains that excessive diagnostic testing may lead to overdiagnosis. In particular, ‘incidental findings’^3^ are considered to be a problematic situation:

And that is, I think, also an ethical dilemma that doctors have, because then you find something and what do I do now? They have no complaints at the moment, so I don’t really have to do something with it now. But imagine that it is cancer, and in four months they will come in with metastatic disease, and then I could have prevented that. That’s difficult. And then the technology renders it unlikely that such a patient says, never mind, we’ll see how things will go. Because everyone says oh, yes, if something can be done about it, then let’s do that scan, then do that biopsy, then do that incredibly complicated procedure.

Incidental findings may be clear instances of pathology, and in these cases, it may be regarded as fortunate that the patient can be treated for a disease that may otherwise have gone undetected until it was too late. However, in other cases incidental findings may be benign deviations or anomalies and it is questionable if the patient will benefit from further diagnostic testing and/or medical intervening, as it is not clear if the anomaly will ever lead to clinical symptoms. Again, this begs the question where to draw the line between normal and abnormal, between health and disease.

#### Problems with translational medicine: from research to the clinic, and beyond

In the interviews, some participants also discussed problems regarding translating medical scientific findings from a research context into clinical practice. One approach that was mentioned by participants as particularly problematic was ‘evidence-based medicine’ (EMB)^4^. While medical professionals may be aware of the different aims and goals of medical research versus clinical medicine, and of the problems surrounding EBM, they may feel bounded by institutional agreements and regulations. For example, insurers may only reimburse treatments that are proven to be effective according to standards of EBM and therefore may not sufficiently allow for tailoring treatment to the personal needs of the patient. HD09 explains how the broad implementation of the EBM style of reasoning, from research to the clinic and beyond, to institutional arrangements, is not without danger:

Evidence-based medicine, with its mono-focus thinking, traditionally, it’s fortunately changing, can also bring real dangers, because what you see is that politics and insurers are very much steering policy and reimbursing on the basis of guideline indicators.

HD13 goes even a step further by provocatively referring to EBM as ‘pharmaceutical-based medicine’. He argues that medical professionals are restricted by the rules and regulations of the healthcare institutions such as the National Healthcare Institute (‘Zorginstituut’), which require treatments to be ‘evidence-based’ before they can be considered eligible for reimbursement. As a result, HD13 claims, we end up with suboptimal medical treatments:

The entire ‘pharmaceutical-based medicine’ is currently ‘the’ steering element of the National Healthcare Institute and of affordable care in the Netherlands, of reimbursed care. And it’s not the best treatment that gets reimbursed, but the treatment that has been the most researched; not the one with the best outcomes.

Another problem that was particularly mentioned in the interviews was that of generalizing medical knowledge from the research context to the clinical context. As diseases and their treatments are commonly researched in study populations that do not represent patient populations in clinical practice - e.g., age range between 18 and 50, mostly Western, male subjects, having only one disease instead of several - a rather homogenous picture of specific disease entities with specific treatments is generated that often does not match the heterogeneous reality in clinical practice. Moreover, while medical research is often focused on curing a disease, or at least reducing its symptoms, patients may in fact have different goals and wishes that need a different approach. Participant HD09 argues that the goals of medical research do not always match the goals of clinical medicine:

So, the average patient in a trial is a middle-aged man. The average user, who is treated according to the guideline based on those trials, is an old woman or one who has more medical conditions and uses several medications. And then it is also the case that those trials are aimed at preventing a new event or surviving. And, for example, not having a second heart attack, not having a stroke. Well, those may be things that are important to someone, but I just said that is often not the most important thing. Those people are not all at about living longer, they care about function preservation. And then it can still be important to prevent that stroke, but then you really have to look at it in a different way.

Especially in case of (chronic) multimorbidity, in which patients suffer from multiple diseases at the same time and also use multiple medications, it can become questionable *what* is treated, exactly. A set of separate diseases, or the combined physiological effects and symptoms of a multitude of underlying pathologies, or even of the medications used? As a consequence, ‘evidence-based’ treatment protocols could potentially harm patient populations that do not fit the assumptions on which the treatment is found to be efficacious. Furthermore, diseases and also the medications that are used may interact, resulting in a clinical picture that is very different from what is expected. We might describe this situation as one that is *epistemologically opaque*: it seems to get very difficult, if not impossible, to distinguish cause and effect. HD09 explains:

And then the question is whether it will work the same way with that woman with all those old age conditions compared to what happened with that fifty-year-old man. So, it probably reacts differently as well. It reacts differently, because there are multiple diseases, interaction with disease. And it reacts differently because there are a whole lot more medications, interacting with medication. And it reacts differently because the body is different.

So, while medical research tries to reduce complexity and look into single homogenous diseases and patient groups, in clinical practice disease often manifests very differently.

#### Communication problems

While participants were generally doubtful about arriving at univocal and all-encompassing definitions of health and disease and favored the idea of conceptual pluralism, some participants also expressed concerns with regard to communication. If we all use different definitions or different health and disease concepts, how do we know we are still speaking of the same thing? As clear-cut definitions are often desired precisely for the purpose of solving ongoing problematic situations in medicine, it may seem paradoxical to accept conceptual pluralism. In practice, having multiple ways to understand a disease can lead to communication problems, participants fear. For example, when medical specialists’ views differ so significantly that they almost literally speak about different diseases, it is questionable if they are still able to sufficiently communicate with each other and their patients.

In an interview with HD08, opposing views on Alzheimer’s Disease among medical specialists were discussed. Alzheimer’s Disease was originally diagnosed on the basis of clinical signs and symptoms, but in recent years a part of the neurologist community has switched to prioritizing biomarker testing (i.e., primarily the presence of beta-amyloid) over clinical presentation. However, the problem is that the group of patients with positive biomarker tests do not completely match the group of patients who get symptoms. Therefore, changing the way of diagnosing Alzheimer’s disease in patients also seem to imply changing the definition of Alzheimer’s disease. Hence, it becomes unclear if medical specialists are still discussing the ‘same’ disease. HD08 says the following about the opposing views:

Well, I think there’s- You could almost say, it’s kind of a clash of civilizations. You have the people who just want a hardcore biological substrate and then have little regard for other aspects. And you have people who say yes, maybe it is not possible to classify it exactly into careful categories, let’s also take into account the less ‘hard’, less definable aspects that are important for the functioning of a patient.

While acknowledging the challenges and pitfalls that come with speaking different ‘medical languages’, at the same time, participants also see benefits of having different approaches towards health and disease. Some of them note that we already are using different languages, scientific explanations and medical classifications, and that this could be viewed as something valuable. In a combined interview with HD13 and HD14, HD14 discusses the different classification systems that are being used for chronic pain patients among different (para)medical professionals:

No, I think you should cherish that, because an anesthesiologist can do things that a rehabilitation doctor cannot do, and vice versa. So, you really have to use each other for that and that also applies to all those other medical specialists and paramedical specialists. So that in itself is not a big deal. What- Or rather, that’s very functional, that’s excellent. At the same time, we must speak each other’s language and that must be the same language with each other, but we must certainly not forget the patient. And, because the patient must also be at the center of our interprofessional communication. And, but also the wishes and needs of the patient. So, if HD13 says ‘I’m good at ICD’, and I’m good at ICF, to put it very bluntly, that’s not going to work. I need to know about ICD, enough to talk to HD13. And HD13 needs to know about ICF, enough to talk with me. But really, we should all be able to know enough to be able to talk to the patient properly.

Thus, interestingly, the suffering of one patient could be classified in several different ways, depending on the classification system that is used. While recognizing the challenges this brings for medical professionals, HD13 and HD14 also see the benefits of looking through different lenses – as long there is sufficient common ground to communicate with each other and the patient. So, concepts of health and disease seems to be approached differently at different levels of care (i.e., primary, secondary, and tertiary lines of healthcare) and between different types of (para)medical professionals. The situation as sketched by HD13 and HD14 seems evident for healthcare as arranged in The Netherlands, where various classification systems are indeed being used in different levels and types of healthcare practices^5^. Every classification system has its strengths and weaknesses. An ongoing challenge seems to lie in being able to sufficiently understand each other’s ‘medical language’.

## Discussion

Philosophers can contribute to medicine by exploring, analyzing and articulating conceptual issues. However, as we take health and disease concepts to be context-dependent, it is crucial to study their meaning in context. Building on recent proposals for a pragmatist understanding of health and disease that embraces conceptual pluralism, investigating different perspectives is very important. As Veit argues: “Questions such as how medical practitioners see, use, and evaluate concepts like health, pathology, and disease are important to the philosophy of medicine. Yet, these questions cannot be answered through introspection alone. They require investigative empirical methods” [21] (p.183). In similar vein, Seidlein & Salloch [17] argue that empirical methods can be used to gain better understanding of the complex relationship between illness and disease, by reflecting upon patient and professional perspectives. Including qualitative methods and other types of empirical research to our toolbox can bring theory and practice closer together and stimulate new medical-philosophical and bioethical explorations.

The current study differs from previous empirical studies [24–27], in that it was specifically focused on exploring how health and disease concepts have a *function* in practice and how they may lead to *problematic situations*. The existing studies have already shown a palette of different conceptualizations, but did not interpret these in terms of their practical function and role in problematic situations. In our interviews, various important practical functions of health and disease concepts were discussed and our participants suggest that different contexts and purposes may require different types of definitions. We agree with Veit that finding such a lack of consensus and a pluralism of concepts and functions, strengthens the case against conceptual monism, and favors positions that “relativise the concept to human interests and cultural dynamics’’ [21] (p.178). Indeed, our study reveals that “the notion [of disease] serves a variety of purposes that perhaps cannot be accomplished using a single concept” [21] (p.180).

However, the plurality of functions and the definitions that are used to serve them, may not always be compatible with each other. A broad concept definition of health may work, for example, to steer healthcare policy in a certain direction on a political or organizational level, but may cause problems when it must be implemented in a research setting. Of course, different functions and definitions do not exist in a vacuum but also interact. Moreover, as is evident from the interviews, although the plurality of definitions may sometimes be problematic for reasons of communication, it is also a reality. Therefore, it may be more fruitful to acknowledge this and to elucidate and explain the differences; this may actually enhance communication and understanding across domains.

In this article, we have highlighted 5 types of problematic situations that were discussed in the interviews and that can be related to the conceptualization of health and disease. Some problems are already recognized in the medical-philosophical literature, such as problem of line-drawing. Others may offer new starting points for medical-philosophical and bioethical inquiry. Philosophy of medicine might help to analyze and elucidate the conceptual components of these problems and come up with suggestions of how conceptual work might help to find solutions. For example, the work that has already been done by Rogers and Walker [12, 31] regarding the line-drawing problem might be useful for medical practitioners and medical guideline developers. They propose using context specific précising definitions that serve to prevent overdiagnosis; such an approach may also be useful to help solve the line-drawing and treatment threshold problems, and the risks of over or undertreatment, that we encountered in this study.

Furthermore, tensions between biomedical and holistic approaches of health and disease – that have led to major debates in the philosophy of medicine and has important ethical implications – were also described by participants as problematic in practice. However, there was also a hint of a solution in the interviews. As one participant explained, different contexts may benefit from different approaches. Strictly biomedical definitions may be more useful for the emergency care doctor while during rehabilitation a holistic normative biopsychosocial model is considered more helpful.^6^ This idea is in line with the proposal by Haverkamp et al. [7], to consider using concepts that fit best with the purposes and values of a specific healthcare practice. Some of the problematic situations described in the interviews may also give new input for investigating these purposes and values in different contexts. For example, the changing conceptualization of Alzheimer’s disease and the use of biomarker diagnostic testing, that was mentioned in the interviews, is a current topic of medical-philosophical and bioethical debate (e.g., see [43–45]).

Another role for philosophy can be to help healthcare professionals and policy makers to better understand how some of their problematic situations arise. For example, some of the issues we identified could be understood in terms of a disconnect between the three spheres of the conceptual triad of ‘disease, illness and sickness’, as originally presented by Twaddle [46] and as later updated by Hofmann [47]. As Hofmann already noted, cases of non-health are generally considered to be less controversial when two or three of the spheres align. However, when only one or two of the are deemed applicable to a certain condition, it becomes epistemically and normatively challenging [47]. This conceptual triad may help patients, healthcare professionals and policymakers to better understand issues around the problem of medically unexplained symptoms, also in relation to the institutional and social function of the disease concept. At this point, it may also be significant to note that in the Dutch language, in which the interviews were conducted, the distinction between disease, illness and sickness is not available. A single word, ‘ziek’ or ziekte’, is used to cover all three notions, making the conceptual confusion perhaps even more salient than in the English-speaking community.

Some of the problematic situations that we have described may, at first glance, be viewed as practical problems with only little conceptual basis. For example, when discussing disease as an institutional and social concept, and describing problems that patients who suffer from medically unexplained symptoms may face (e.g., problems with accessing certain healthcare resources, or social and financial arrangements), one might question to what extent this is a problem with the conceptualization of disease.

One might argue, as Hesslow [48] did, that we have been misled by the idea that we need a concept of disease to make normative decisions on clinical, moral or socially important issues. However, from a pragmatist perspective, the theoretical, practical and normative dimensions of concepts are inherently related. As De Vreese argues: ‘‘it seems undeniable that the health/disease distinctions made on the basis of tacit understandings of the disease notion do play an important role in the background of health care-related research and decision-making processes (clinical, moral, legal, social, or otherwise), which might have important consequences in practice’’ [6] (p.429). Starting from this observation, we might consider adapting our concepts to better fit the social and institutional arrangements (cf. [49, 50]). or we might propose better concepts or criteria to base these decisions on (e.g., see [51]). Both seem to be pre-eminently tasks for philosophers and ethicists to pursue. Additionally, empirical studies may help to further explore these ‘tacit understandings of the disease notion’ and investigate what these ‘important consequences in practice’ entail, as starting points for further philosophical and ethical reflection.

### Limitations

As is common for qualitative research, results cannot be generalized and results may not represent the views, attitudes and beliefs of the whole community of medical professionals or patient organizations. As the sample of this study is relatively small and consisted of a broad range of professionals, the findings should be viewed as starting points for further investigation, not definitive answers. Moreover, as indicated in the methods section, the sample consisted of a group of highly educated and experienced professionals. Although there were good reasons to select them, it is important to remark that as a consequence, we did not study the views and experiences of other, more ‘ordinary’ healthcare workers and patients. Also, we did not include the views of different nationalities, cultures, and/or for example less educated or marginalized people. Indeed, we should ask: ‘who are the rightful owners of the concepts disease, illness and sickness’ [9]? If we view health and disease as plural concepts then an empirical philosophy of medicine should do justice to this plurality by including the views and experiences of these groups as well. Future studies may focus on investigating more specific groups (e.g., a specific medical specialist field or certain group of patients) and/or institutional contexts.

Furthermore, as we have learned from discussions on the empirical turn in medical ethics [52], one should be careful and considerate when making normative claims on basis of empirical data. However, given the explorative character of this study, this is not deemed a significant problem. Our aim was to explore the range of views regarding health and disease concepts, and the existence of problematic situations related to health and disease concepts, not to give an exhaustive or quantitative overview of such concepts and situations. Furthermore, in qualitative research, it is generally acknowledged that the researcher is not merely a ‘neutral observer’ but also an actor who actively engages with participants in the research process, and thus, is part of the data that is generated [53]. In this study in particular, with its aim of exploring how health and disease concepts function in practice and examining whether they could lead to problems, the interview guide was drafted from a specific theoretical angle. Moreover, the interviews were analyzed with existing theoretical discussions and frameworks in the back of our minds. By being open and reflexive about this process, and by making our interpretations as transparent as possible, we hope to have gained sufficient rigor.

## Conclusion

The traditional debate on health and disease concepts commonly departs from theory rather than from practice. In line with recent calls for experimental philosophy of medicine and empirical philosophy of science, we suggest that theoretical work could benefit from incorporating empirical research. In this qualitative interview study, we have examined the relevance and significance of health and disease concepts, as experienced by participants in various healthcare practices. We found that there are three types of functions that health and disease concepts serve in practice: (1) ‘Strategic development, politics and policy-making’, (2) ‘Institutional and social function’, and (3), ‘Guiding clinical practice and medical research’. Being aware of these different purposes may prevent bluntly using concepts beyond their functional scope. We also explored what kind of difficulties participants experienced in relation to the conceptualization of health and disease in practice, and found five main types of problematic situations: (1) Illness without identifiable pathology, (2) Biomedical versus holistic approaches, (3) Line-drawing and treatment threshold problems, (4) Problems with translational medicine: from research to the clinic, and beyond, and (5), Communication problems.

This study demonstrates how concepts of health and disease can influence different aspects of healthcare and healthcare-related practices and may sometimes contribute to complex problematic situations. By analyzing these influences, by making underlying implicit assumptions explicit, giving further interpretation to the problems observed in practice, providing theoretical frameworks and conceptual tools, and by suggesting conceptual changes or adaptations, we might be able to help solve some of these problems. To do this in a proper way, we need both theoretical and empirical work. If we want our philosophical definitions to be a part of the solution for real-world problems, it is important to consider the intuitions and ideas of people working in different types of medical fields, patients, researchers, and all other stakeholders [20]. Paraphrasing Immanuel Kant, we may conclude that philosophy of medicine without empirical research risks being empty, while empirical research without philosophical theorizing will still leave us blind. Going back and forth between theory and practice will probably result in a more complex but hopefully also in a better and more fruitful understanding of concepts of health and disease.

### Electronic supplementary material

Below is the link to the electronic supplementary material.

Supplementary Material 1

## Data Availability

The data that support the findings of this study are available from the Erasmus Medical Center but GDPR restrictions apply to the availability of these data and are therefore not publicly available.

## Footnotes

1 The notion of experimental philosophy is relatively new and its definition is therefore not yet solidified. Sometimes it is used broadly, including various kinds of empirical research methods. In other instances, it refers specifically to philosophical studies with an experimental design, in which one variable is changed in isolation to measure changes in a philosophically relevant outcome (e.g., moral judgement). We believe that the latter, more narrow definition is useful to distinguish between experimental and other empirical studies. Therefore, in the title of our study, we explicitly use the term empirical philosophy instead of experimental philosophy.

2 This is, at least, how things are arranged in the Netherlands. Similar arrangements are in place in many other countries worldwide.

3 Incidental findings are anomalies that are detected in clinical tests that were in fact aimed on testing something else. As the clinical significance of these findings is often not clear, clinicians and/or clinical researchers are confronted with ethical dilemmas [37–39].

4 EBM can be described as an approach towards medicine that takes scientific evidence as a central point for guiding clinical decision-making. Typically, in EBM meta-analyses and randomized clinical trials (RCTs) are considered to be the highest forms of scientific evidence. While these methods can indeed have strong benefits over other types of medical research, there is ample discussion about its down sides as well [40–42].

5 For instance, general physicians, who provide primary care, use a different classification system (International Classification of Primary Care; ICPC) than a medical specialist in a hospital (International Statistical Classification of Diseases and Related Health Problems; ICD), who provides secondary and tertiary care, uses. Physiotherapists (International Classification of Functioning, Disability and Health; ICF), and psychologists (Diagnostic and Statistical Manual of Mental Disorders; DSM), in turn, also use different types of classification systems.

6 Another way to frame this would be to say that in emergency care, only ‘disease’ may be relevant to provide proper medical care, whereas in a rehabilitation setting the whole triad of disease, illness and sickness is being addressed.
