# Supplementary material for: Exploring health and disease concepts in healthcare practice: an empirical philosophy of medicine study
Source: BMC Med Ethics. 2024 Mar 27;25:38. doi: 10.1186/s12910-024-01037-9 (PMC10967067; doi:10.1186/s12910-024-01037-9)
Supplement: Supplementary file 1 — Supplementary Material 1 [file 12910_2024_1037_MOESM1_ESM.docx]

**Appendix**

1. Interview questions (semi-structured)

***Introduction interview***

- How would you define the terms 'health' and ‘disease’ yourself? Would colleagues in your field agree with your definitions?
- What are the views on ‘health’ and ‘disease’ within your field of expertise? Are these concepts defined in any particular way?
- What are these concepts for, practically speaking, in your field of work/organization/et cetera?
- Are there any other terms used other than ‘health’ and ‘disease’?
- Why are these terms specifically used? What is the advantage of using these concepts over the concepts of 'disease' and 'health'?
- Are there different definitions that are used side by side?
- Do you think it is useful to have definitions of health and disease that are all-encompassing? Why (not)? Do you think it is possible to formulate such definitions? Why do you think that?
- What are the consequences of granting the status healthy/diseased for the individual? And for the organization? Is this label necessary, for example, to start a certain process?
- Can you give me an example of how ‘health’ and ‘disease’ is normally determined within your field of work/organization/et cetera?

***Explanation of problematic situation***

I will now give you a brief explanation of what we call a ‘problematic situation’. By a problematic situation we mean a situation in which current views/definitions of health and/or disease are no longer sufficient for the continuation of a certain activity or the achievement of a certain goal. Previously used definitions, for example, are no longer sufficiently useful to achieve the goals within your domain/field/profession. Another possibility is that there are conflicting values/opinions about health and disease, which causes problems in terms of communication or problems in a certain activity. In philosophy, a problematic situation is sometimes characterized as a starting point to search for a new conception or definition.

- Are there any specific problematic situations that you encounter in practice that are related to definitions of health and disease?
- Can you give practical examples in which such problematic situations occur?
- Do you see any solutions to such problematic situations or have there already been solutions brought forward to solve these issues?
- If not, do you have your own ideas on how to solve these problems in relation to the disease and health concepts or how to improve the situation?

1. List of study participants their expertise/background (in random order)

| Strategic advisor at a university medical center, educational coordinator, PhD in philosophy |
| --- |
| Public health consultant, philosopher, moral case deliberation facilitator |
| Professor of geriatrics, medical specialist internal medicine and geriatrics |
| President patient organization, professor of revalidation medicine, physiotherapist |
| Representative patient organization, policy advisor, teacher in nursing, ex-military nurse |
| Associate professor of public health, medical specialist social medicine |
| General physician, ethicist, president patient organization |
| President patient organization |
| Healthcare researcher, PhD in philosophy |
| Professor of neurology, neurologist |
| Specialist internal medicine and geriatrics, medical ethicist (PhD) |
| Philosophy consultant, insurance doctor (not practicing) |
| Professor of family medicine, general physician |
| Medical doctor, specialist occupational medicine |
| President patient organization, professor of pain and palliative medicine, anesthesiologist |
| Insurance doctor, PhD in cardiology |
| Representative patient organization, policy advisor |

The participants in this study are all highly educated/trained and experienced professionals in their respective fields. We aimed for recruiting a broad range of professionals, with different backgrounds, and working in a variety of fields and organizations. The participants did not receive financial reimbursement and there were no conflicting interests between the researchers and the participants.

1. Context and background of the research(ers)

The qualitative study is embedded in a larger research project which aims for exploring a pragmatist perspective towards the conceptualization of health and disease. In a previous publication by us, we have suggested that the use of empirical studies, as well as historical analyses, may improve and refuel the debate on health and disease concepts. RL is a PhD candidate in the philosophy of medicine, (with an MA in bioethics and philosophy and an MSc in clinical psychology) supervised by MS, who is a professor in philosophy of medicine and medical ethics (a former MD, MA philosophy and PhD in medical ethics). The interviews were conducted by RL; the analysis and writing was done in good collaboration between both researchers. RL has received training in qualitative methods during his PhD trajectory. MS is experienced with conducting qualitative studies and supervised RL in designing and conducting the study.
